# *In silico* simulation of future hybrid performance to evaluate heterotic pool formation in a self-pollinating crop

**DOI:** 10.1038/s41598-020-61031-0

**Published:** 2020-03-04

**Authors:** Wallace A. Cowling, R. Chris Gaynor, Roberto Antolín, Gregor Gorjanc, Stefan M. Edwards, Owen Powell, John M. Hickey

**Affiliations:** 1 The UWA Institute of Agriculture, and UWA School of Agriculture and Environment, The University of Western Australia, Perth, WA 6009 Australia; 2 Roslin Institute and Royal (Dick) School of Veterinary Studies, University of Edinburgh, Easter Bush Research Centre, Midlothian, EH25 9RG UK

**Keywords:** Heritable quantitative trait, Plant breeding

## Abstract

Hybrid vigour has the potential to substantially increase the yield of self-pollinating crops such as wheat and rice, but future hybrid performance may depend on the initial strategy to form heterotic pools. We used *in silico* stochastic simulation of future hybrid performance in a self-pollinating crop to evaluate three strategies of forming heterotic pools in the founder population. The model included either 500, 2000 or 8000 quantitative trait nucleotides (QTN) across 10 chromosomes that contributed to a quantitative trait with population mean 100 and variance 10. The average degree of dominance at each QTN was either 0.2, 0.4 or 0.8 with variance 0.2. Three strategies for splitting the founder population into two heterotic pools were compared: (i) random split; (ii) split based on genetic distance according to principal component analysis of SNP genotypes; and (iii) optimized split based on F_1_ hybrid performance in a diallel cross among the founders. Future hybrid performance was stochastically simulated over 30 cycles of reciprocal recurrent selection based on true genetic values for additive and dominance effects. The three strategies of forming heterotic pools produced similar future hybrid performance, and superior future hybrids to a control population selected on inbred line performance when the number of quantitative trait nucleotides was ≥2000 and/or the average degree of dominance was ≥0.4.

## Introduction

The value of heterosis, or hybrid vigour, has been captured and exploited effectively in maize, rice, sorghum, pearl millet, rye, oilseeds, cotton, sunflower, sugar beet, trees, vegetable crops, and ornamentals^1–4^. Global increases in grain production of 15 to 50% occurred when hybrids replaced superior open-pollinated varieties in maize, sorghum, sunflower and rice^5^. In self-pollinating species such as wheat, where heterosis is typically low (10%) and commercial adoption of hybrid wheat is low, there is an incentive to increase yield and yield stability through hybrid breeding, and reciprocal recurrent genomic selection (RRGS) was advocated as a way to improve hybrid breeding in that crop^6^.

Recent molecular genetic studies support the dominance theory of heterosis in rice^7^ and maize^8^, and while epistasis may occur, it is not a significant contributor to heterosis^9,10^. Average dominance in maize was less than unity^9,11^, which confirmed that dominance, not overdominance, was the major contributor to heterosis in maize. However, pseudo-overdominance due to linkage can also occur^11,12^ and is impossible to distinguish from true overdominance^9,10^. Lamkey and Edwards^13^ provide theoretical quantitative genetics support for directional dominance and differences in allelic frequency between two populations as major contributors to panmictic-midparent heterosis, particularly in self-pollinating crops where inbreeding depression is minimal.

Hybrid breeders in maize and other cross-pollinating crops have maximised heterosis through the development of heterotic groups by reciprocal recurrent selection (RRS). Maize heterotic groups developed from open-pollinated Corn Belt Dent varieties^14^. The Iowa Stiff Stalk Synthetic, Non-Stiff Stalk, and Iodent heterotic groups diverged from a relatively homogeneous source^15,16^. Tracy and Chandler^17^ concluded that maize heterotic groups in the USA were formed by trial and error from a single race of corn, and that genetic divergence between heterotic groups is a natural consequence of RRS. This conclusion is supported by SNP marker evaluation of RRS in the U.S. Department of Agriculture’s RRS experiment between the Iowa Stiff Stalk Synthetic (BSSS) and the Iowa Corn Borer Synthetic No. 1 (BSCB1) populations^8^. Rapid genetic divergence occurred between the BSSS and BSCB1 populations during 16 cycles of RRS as shown by a genome-wide panel of 40,000 SNP markers^8^.

The criteria for grouping of founders into heterotic pools are particularly important in self-pollinating crops, where there is little or no history of hybrid breeding or previous development of heterotic pools. Information on the relative efficacy of different methods of heterotic pool formation is scarce. This has stimulated research on methods to establish heterotic pools at the very beginning of hybrid breeding^18,19^. Melchinger^20^ argued that mean heterosis and hybrid performance in intergroup crosses would be maximized by increasing the difference in allele frequencies between the two subgroups; and secondly that heterotic patterns may be identified on the basis of hybrid performance in diallel or factorial crosses among members of the population. Another approach is a genome-based strategy to identify heterotic groups based on general combining ability (GCA) among the base population^19^. Melchinger and Gumber^21^ proposed a multi-stage procedure for establishing heterotic pools, where groups of individuals are initially separated on genetic similarity followed by production and evaluation of testcrosses.

*In silico* models for simulation of genomic selection in crop breeding are available^22^ and have been used to evaluate various methods of integration of genomic selection into traditional wheat breeding^23^ and hybrid wheat breeding^6^. Simulation based on pedigree or genomic relationship information allows the evaluation of long-term outcomes of strategies implemented early in the breeding process, such as the use of optimal contributions selection and optimal mating designs to replace truncation selection^24,25^.

The objective of this paper was to use stochastic simulation in a self-pollinating crop to compare three strategies of forming heterotic pools on future hybrid performance - random separation of founder lines into two heterotic pools, separation based on genetic distance among founder lines, and separation based on initial F_1_ hybrid performance in a diallel cross among the founders. Our hypothesis was that random splitting of founders would be inferior to separation based on genetic distance or initial F_1_ hybrid performance in a diallel cross.

We used stochastic simulation of future hybrid performance over 30 cycles of RRS to compare the three strategies of heterotic pool formation. RRS occurred against the opposite heterotic pool, with selection based on GCA for true genetic value (TGV) of hybrids against the opposite pool (RRS-TGV). We assume that heterosis in a self-pollinating crop is panmictic-midparent heterosis which depends only on genetic divergence and degrees of dominance, and is not affected by inbreeding depression^13^. Therefore, we evaluated a range of quantitative trait nucleotides (QTN) controlling the quantitative trait, and a range of degrees of dominance at QTN (from 0.2 to 0.8), which represented the range of degrees of dominance expected in maize^9,11^ and in self-pollinating plants^7,26^.

We model TGV of individuals as the sum of genetic values at hypothetical QTN across the genome, as we have done previously (23, 25). However, our estimate of TGV is not subject to environmental error, and we minimise genetic drift by modelling with TGV on large numbers of progeny. This allows us to focus on answering the key question: “in the absence of environmental error and with minimal genetic drift, what is the impact of method of heterotic pool formation in a self-pollinating crop on future hybrid performance?”.

The three strategies of forming heterotic groups from founder lines were assessed for future hybrid performance under RRS-TGV, and compared to a control population which underwent *per se* selection followed by hybrid formation among superior inbred lines.

## Results

### Impact of method of heterotic pool formation on future hybrid performance

**Figure 1:**
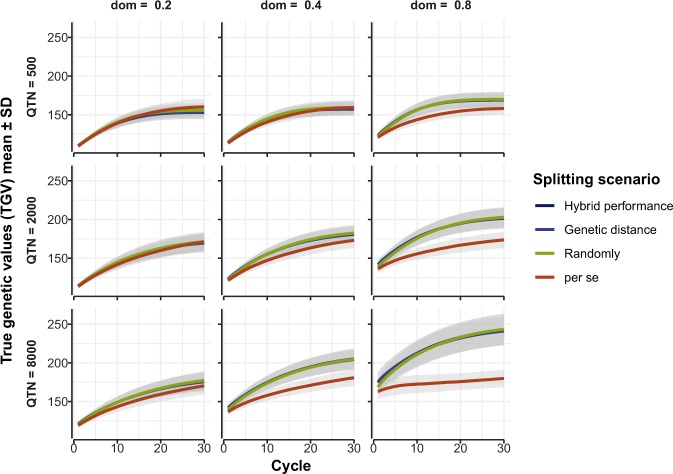
Predicted future F_1_ hybrid performance for a quantitative trait under three scenarios of heterotic pool formation in a self-pollinating crop. Mean true genetic values (TGV) ± standard deviation (SD) (shading) from 20 runs of the model are shown on the vertical axes for three methods of splitting the founder population into two heterotic pools (“Hybrid performance”, “Genetic distance” and “Randomly”) followed by RRS-TGV for 30 cycles, compared with “*per se*” selection in a control population. The models were repeated for three levels of quantitative trait nucleotides (QTN = 500, 2000, 8000) and three levels of average degree of dominance (dom = 0.2, 0.4 and 0.8).

The simulations showed no difference in future hybrid performance over 30 cycles of RRS-TGV for the three strategies of splitting the founder population into heterotic pools (Fig. 1). The control population, selected for *per se* performance of inbred lines, produced F_1_ hybrids equal in average performance to those selected by RRS-TGV when the number of QTN was less than 2000 and/or the average degree of dominance was less than 0.4 (Fig. 1). When the number of QTN exceeded 2000 and/or the average degree of dominance exceeded 0.4, F_1_ hybrids selected by RRS-TGV were superior to hybrids formed in the control group (Fig. 1).

### Impact of method of heterotic pool formation on future line performance

**Figure 2:**
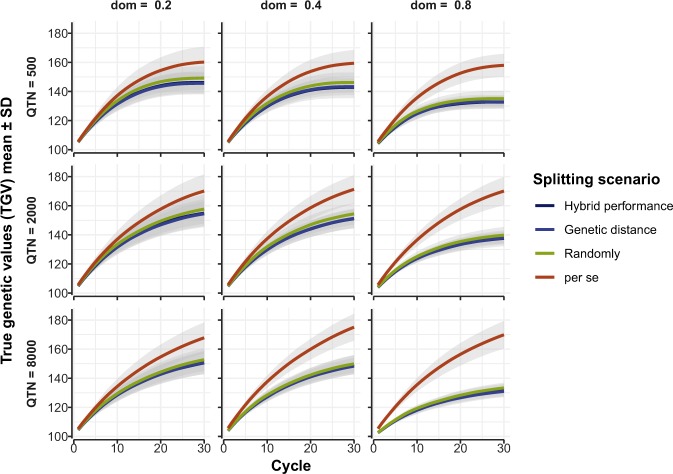
Predicted future inbred line performance for a quantitative trait under three scenarios of heterotic pool formation in a self-pollinating crop. Mean true genetic values (TGV) ± standard deviation (SD) (shading) from 20 runs of the model are shown on the vertical axes for three methods of splitting the founder population into two heterotic pools (“Hybrid performance”, “Genetic distance” and “Randomly”) followed by RRS-TGV for 30 cycles, compared with “*per se*” selection in a control population. The models were repeated for three levels of quantitative trait nucleotides (QTN = 500, 2000, 8000) and three levels of average degree of dominance (dom = 0.2, 0.4 and 0.8).

The three strategies for splitting the founder population into heterotic pools gave similar average line (inbred) performance over 30 cycles of RRS-TGV, and line performance in heterotic pools was always less than in the control population (Fig. 2). Line performance with 500 QTN reached a plateau at 20 to 25 cycles of RRS-TGV, but the plateau was delayed with 2000 or 8000 QTN (Fig. 2).

### Impact of method of heterotic pool formation on variance of true genetic values

**Figure 3:**
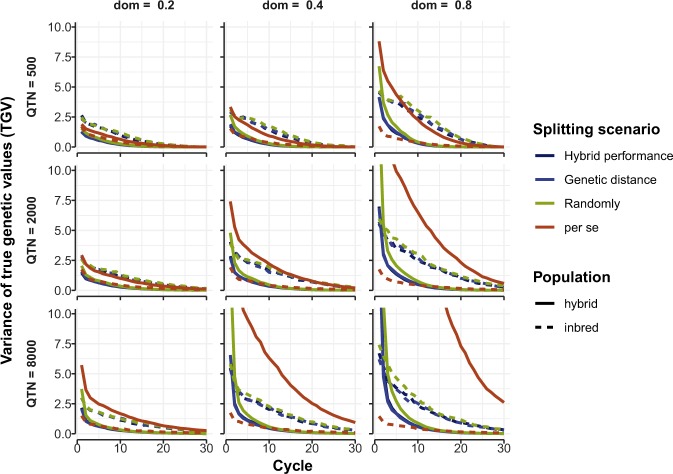
Variance of true genetic values (TGV) for a quantitative trait under three scenarios of heterotic pool formation in a self-pollinating crop. Mean genetic variance of hybrids (solid lines) and inbred lines (dashed lines) from 20 runs of the model are shown on the vertical axes for three methods of splitting the founder population into two heterotic pools (“Hybrid performance”, “Genetic distance” and “Randomly”) followed by RRS-TGV for 30 cycles, compared with “*per se*” selection in a control population. The models were repeated for three levels of quantitative trait nucleotides (QTN = 500, 2000, 8000) and three levels of average degree of dominance (dom = 0.2, 0.4 and 0.8).

Genetic variance dropped rapidly during 30 cycles of RRS-TGV in both F_1_ hybrids (solid lines, Fig. 3) and inbreds (dashed lines, Fig. 3). Genetic variance among inbreds in RRS-TGV was always greater than the genetic variance of inbreds in the control population. The reverse was true for genetic variance in F_1_ hybrids: the genetic variance among hybrids formed during 30 cycles of *per se* selection was always greater than in any method of heterotic pool formation, especially when the number of QTN was greater than or equal to 2,000 and dominance degrees greater than or equal to 0.4 (Fig. 3).

### Impact of method of heterotic pool formation on molecular genetic diversity

**Figure 4:**
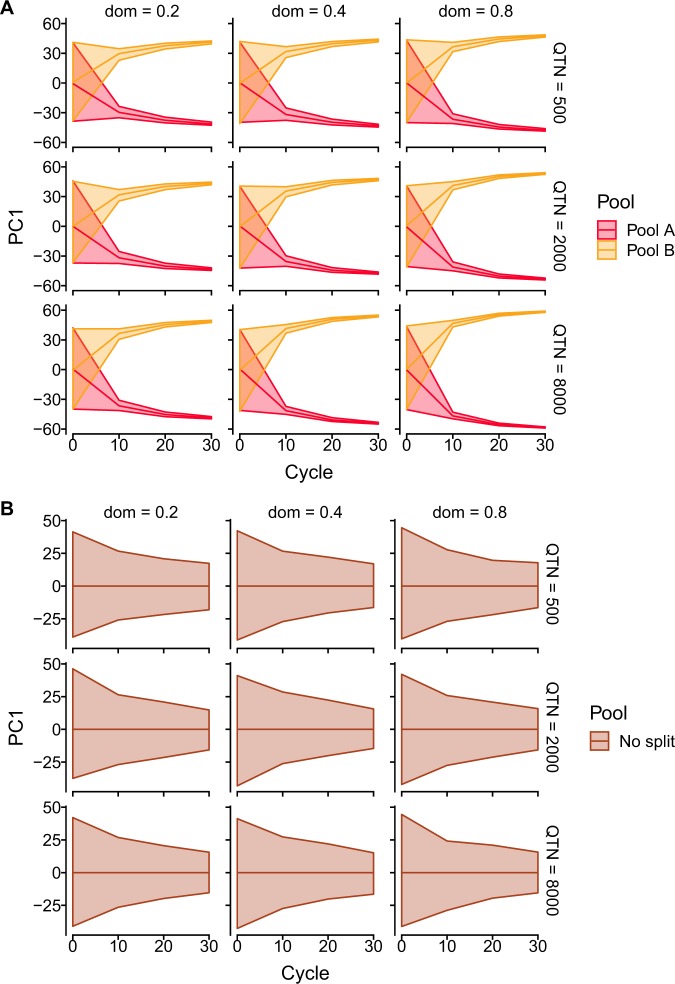
The average (middle line), minimum (lower line) and maximum (upper line) of SNP-based genetic diversity from 20 runs of the model over 30 cycles, based on the first axis (PC1) of principal co-ordinate analysis of SNP genetic diversity among individuals when the founder population is (**A**) randomly split into two heterotic pools (pools **A** and **B**) and subject to reciprocal recurrent selection, or (**B**) not split into pools and subjected to *per se* selection only. The models were repeated for three levels of quantitative trait nucleotides (QTN = 500, 2000, 8000) and three levels of average degree of dominance (dom = 0.2, 0.4 and 0.8).

The SNP molecular genetic diversity in the heterotic pools decreased more rapidly during RRS-TGV (Fig. 4A) than in the control population where inbred lines were selected for *per se* performance (Fig. 4B). This reduction in SNP diversity diversity in heterotic pools occurred rapidly during the first 10 cycles of RRS-TGV, and heterotic pools were strongly separated based on SNP diversity after 30 cycles of RRS-TGV (Fig. 4A). Similar outcomes were obtained for each method of heterotic pool formation and each level of QTN and degrees of dominance (Figs. 4A, S1A,B), and for each PCA axis (data for PC2 not shown).

## Discussion

We compared three strategies for splitting founders of a hypothetical self-pollinating crop into heterotic pools at the start of reciprocal recurrent selection, based on *in silico* stochastic simulation of future hybrid performance for a quantitative trait. The future performance of hybrids was similar when the founder population was split into heterotic pools based on the basis of molecular genetic distance^18,20^, or based on GCA in a simulated half-diallel cross among the founders^27^. Random splitting of founder lines into heterotic pools performed equally well to any other method of splitting based on future hybrid performance. Our results support the conclusion that arbitrary splitting of inbreds into two heterotic groups is a valid method to begin hybrid breeding in self-pollinating crops^17^.

We base our stochastic simulation of F_1_ hybrid breeding on the dominance model of heterosis^9^, where the major contributing factor to hybrid vigour is the degree of dominance at loci contributing to grain yield. While no single model of heterosis fully explains empirical observations, support for the dominance model of heterosis was provided theoretically by Lamkey and Edwards^13^, and incomplete dominance explained heterosis in the molecular genetic dissection of maize hybrids^8^ and rice hybrids^7^. Potentially, polyploidy may increase the opportunities for complex epistasis through interlocus and intergenomic interactions, but epistasis was not a major cause of heterosis in rice^7^. Our *in silico* simulation of the dominance model of heterosis in a hypothetical self-pollinating species supports the conclusion that arbitrary splitting of inbreds into two heterotic groups is a valid method to begin hybrid breeding in self-pollinating crops^17^.

The range of degrees of dominance used in this study (0.2 to 0.8) represents the range of dominance expected in most crops. Crow^9^ and Moll *et al*.^11^ report incomplete dominance for grain yield between 0.5 and 1.0 in maize. In self-pollinating plants, the average degree of dominance for grain yield was less than 0.5^7,26^. In our simulations, the best outcome for breeding with heterotic pools occurred when average degrees of dominance was 0.8, and there was no advantage of RRS-TGV over *per se* selection (line breeding) for future hybrid performance when average degrees of dominance was 0.2.

In our simulations, the number of QTN for the hypothetical quantitative trait across the genome varied from 500 to 8000. Significant advantage was apparent with 8000 QTN in terms of future hybrid performance. This reflects the importance of high allelic diversity in the founder population for future hybrid performance.

In summary, our simulations demonstrated an advantage for future hybrid performance when selection was based on RRS-TGV in heterotic pools compared with *per se* selection, but only when there was high allelic diversity (number of QTN ≥ 2000) and/or high dominance at loci (≥0.4) for a quantitative trait. However, there was no difference between any method of splitting the founder population into heterotic pools in terms of future hybrid performance – splitting based on SNP-based genetic distance or GCA from a diallel cross among founders produced the same result as random splitting. In other words, the process of reciprocal recurrent selection drove the heterotic pools apart, and this overwhelmed any potential advantage of splitting founders based on genetic distance or GCA from a diallel cross.

RRS-TGV was superior to *per se* selection for future hybrid performance when the degrees of dominance was ≥0.4 and there were large numbers of QTN (≥2000) affecting the trait. The “cost” of RRS-TGV was a rapid decline in SNP genetic diversity within heterotic pools over 30 cycles of RRS-TGV (Figs. 4A, S1A,B). This may be justified by the production of superior hybrids when there is high allelic diversity and/or high dominance at loci for a quantitative trait.

It is important to emphasise that the stochastic simulation of RRS-TGV in this paper is not equivalent to simulation of commercial hybrid breeding. We do not simulate genotype by environment interaction and we use a very large population size to minimise genetic drift. These assumptions allowed us to focus on the comparison between the three strategies for formation of heterotic pools in a self-pollinating crop. Our results support the conclusions of Tracy and Chandler^17^ that the initial separation of inbreds into heterotic pools may be done arbitrarily. It would be interesting in future to expand the simulation to include conditions relevant to commercial hybrid breeding. One result of our research is that high numbers of QTN and high average dominance degrees are important for future hybrid performance, but achieving this result in practice will be a challenge in self-pollinated crops.

To achieve high levels of genetic diversity (high numbers of QTN) for the quantitative trait in the founder population, breeders should consider base-broadening in self-pollinating crops^28–30^ before forming heterotic pools and starting RRS. Breeders should also consider conserving population genetic diversity during RRS through the use of optimal contributions selection^22,24,25,31^. However, new research will be necessary to enable an increase in the degree of dominance at individual trait-related loci.

## Materials and Methods

We used *in silico* stochastic simulation to evaluate three strategies of heterotic pool formation in a self-pollinating crop. The simulations were performed in R^32^ using the package AlphaSimR^23^ and 20 replicates were performed in each strategy.

In each replicate, we first simulated 100 genetically unique inbred founders, and this group of founders was used in each of the three strategies of heterotic pool formation and a control population. For each strategy of heterotic pool formation, simulation of future breeding cycles (described below) occurred over 30 cycles of reciprocal recurrent selection based on TGV of hybrids formed against elite lines in the opposite pool (RRS-TGV). In the control population, simulation occurred over 30 cycles of selection based on TGV of inbred lines (*per se* selection), and future hybrid performance was assessed on hybrids among selected inbred lines. We report the mean and standard deviation of TGV from 20 replicates in each cycle for both inbred lines and hybrids in each strategy of heterotic pool formation and the control population.

### Simulation of founders

The simulation of founders was based on coalescent theory as modelled by the Markovian Coalescent Simulator (MaCS)^33^. A unique founder population of 100 genetically diverse inbred plants was simulated for each replicate. Sequences for each plant were generated in AlphaSimR by a call to MaCS^33^. The genome of the simulated plants consisted of 10 chromosome pairs in a model crop species which is self-pollinated and capable of doubled haploidy. Each chromosome was assigned a genetic length of 1 Morgan and a physical length of 10^8^ base pairs. Recombination rate was 10^−8^ per base pair, and mutation rate was set to 2.5 × 10^−8^ per base pair. Effective population size was set to 100 at 10 cycles prior to formation of the founder population. Sites segregating in the founders’ sequences were then randomly selected to serve as 10,000 single nucleotide polymorphisms (SNP) and 500, 2000 or 8000 QTN with a restriction that there was an equal number of SNP and QTN per chromosome. No population structure was imposed during the simulation of founders. The degree of genetic diversity over cycles of selection was reported in each population based on the diversity accounted for in the first axis of principal co-ordinate analysis of SNP genetic diversity among individuals.

A quantitative trait that could be representative of yield was simulated for each scenario by randomly assigning additive and dominance effects to the predetermined, biallelic QTN. The magnitudes of these effects were scaled in the founders to ensure that the average genetic merit for the trait, or average TGV, was 100 with variance of 10. This was accomplished by sampling additive effects from a standard normal distribution and degrees of dominance from a normal distribution with a mean of 0.2, 0.4 or 0.8 and a variance of 0.2 for all QTN. We consider only incomplete dominance, where degrees of dominance is less than unity, that is, less than the additive effect^34^. Degrees of dominance were converted to dominance effects by multiplying them with the absolute value of its corresponding additive effect. The additive effects, and thereby the dominance effects too, were then linearly scaled to achieve the desired genetic variance of 10. The desired mean was then achieved by adding a fixed value to the TGV of all individuals.

### Future hybrid performance

The three strategies for forming heterotic groups were evaluated based on future hybrid performance. The three methods for splitting founders into heterotic pools were (i) split randomly; (ii) split based on genetic distance from principal component analysis of genotypes; (iii) optimized split based on GCA, that is, F_1_ hybrid performance in a simulated diallel cross among the 100 founders. Future hybrid performance was evaluated over 30 cycles of RRS-TGV where parents for the next cycle were selected on the basis of their GCA against elite lines in the opposite pool. A control population was not separated into heterotic pools, but selected on the basis of TGV of inbred lines (*per se* selection) in each cycle. In both RRS-TGV and *per se* selection, we report future hybrid performance among selected parent lines. Each scenario involved the same total number of individuals (1000), and same selection proportion (10%) in each cycle.

### Strategies for splitting the founder population into heterotic pools

#### Randomly

To begin cycle 1, the 100 founders were split randomly into two pools of 50 (pools A and B), and 500 random matings were made within pools to produce one doubled haploid (DH) progeny per mating. Selection was based on GCA performance against the opposite pool. In pool A, 500 cycle 1 DH progeny were crossed with the 50 parent lines in pool B, and the GCA of each pool A line was calculated from the sum of QTN effects. The mean and variance of TGV of F_1_ hybrid performance in pool A was reported. The top 50 (10%) of pool A individuals was selected based on GCA against pool B, and intercrossed to begin cycle 2 by making 500 random matings among them. One DH progeny was produced per mating, and the mean and variance of TGV of 500 cycle 2 lines in pool A was recorded. The same process was followed in pool B: 500 cycle 1 DH progeny were crossed with the 50 parent lines from pool A, and the top 50 (10%) of pool B individuals was selected for GCA against pool A to begin cycle 2. The mean and variance of inbreds in pools A and B was averaged, and this average was used in graphs of inbred performance and variance across cycles. RRS-TGV continued in this fashion over 30 cycles.

#### Genetic distance

To begin cycle 1, the 100 founders were split into two pools of 50 using the first principal component of their SNP data. The process of selection in each cycle from thereon was identical to that used in (i), starting with 500 random matings within pools that produced one DH progeny per mating, and with selection based on GCA performance against the opposite pool.

#### GCA in a simulated diallel cross among the founders (“hybrid performance”)

To begin cycle 1, F_1_ hybrids were formed from a full diallel of the 100 founders, and the founders were split into two groups of 50 (pools A and B) to maximise the between-pool F_1_ hybrid performance. The optimal split was determined using the genetic algorithm presented in Ladejobi *et al*.^35^ which maximises the mean of hybrids in crosses between the two pools. The process of GCA selection in each cycle from thereon was identical to (i), starting with 500 random matings within pools and one DH progeny per mating, and with selection based on GCA performance against the opposite pool.

### Control population

The control population was not separated into heterotic pools, but selected on the basis of TGV of inbred lines (*per se* selection) in each cycle. This began in cycle 1 with 1,000 random matings among 100 founders, with 1 DH progeny per mating. For each progeny, the value of QTN effects was summed across all QTN. DH progeny (lines) were ranked on the basis of TGV, and the mean and variance of lines reported in each cycle. The top 10% (100) DH progeny were promoted and 1,000 random matings were made among them, with one DH progeny per mating, to begin the next cycle. F_1_ hybrid performance was evaluated by making F_1_ hybrids in a half-diallel of the top 100 DH selections, excluding selfs, and reporting the mean and variance of F_1_ hybrid performance in each cycle.

## Supplementary information

Supplementary information.
