# Supplementary material for: In silico simulation of future hybrid performance to evaluate heterotic pool formation in a self-pollinating crop
Source: Sci Rep. 2020 Mar 4;10:4037. doi: 10.1038/s41598-020-61031-0 (PMC7055256; doi:10.1038/s41598-020-61031-0)
Supplement: Supplementary file 1 — Supplementary information. [file 41598_2020_61031_MOESM1_ESM.pdf]

Supplementary File 1

In silico simulation of future hybrid performance to evaluate heterotic pool formation in a self-pollinating crop

Wallace A. Cowling*, R. Chris Gaynor, Roberto Antolín, Gregor Gorjanc, Stefan M. Edwards, Owen Powell and John M. Hickey

Figs. S1A and S1B

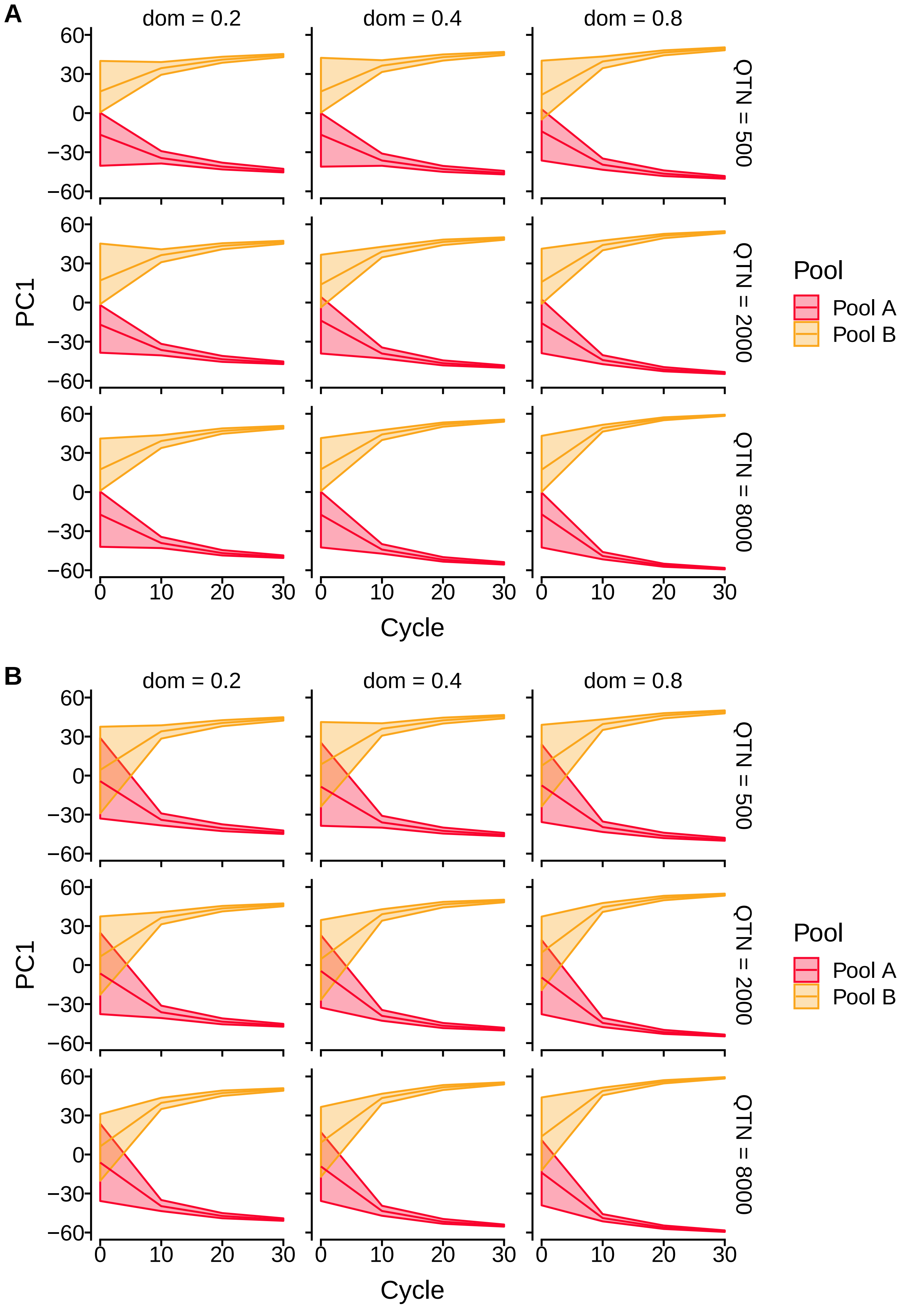

A
B
dom = 0.2
dom = 0.4
dom = 0.8
QTN = 500
QTN = 2000
QTN = 8000
PC1
Cycle
Pool
Pool A
Pool B
